# Lewis b antigen is a common ligand for genogroup I norovirus strains

**DOI:** 10.1002/2211-5463.13455

**Published:** 2022-07-04

**Authors:** Yuichi Someya

**Affiliations:** ^1^ Department of Virology II National Institute of Infectious Diseases Musashi‐Murayama Japan

**Keywords:** histo‐blood group antigens, Lewis antigens, norovirus, virus‐like particle

## Abstract

Noroviruses are major causative agents of nonbacterial acute gastroenteritis in humans. Ten genogroups of noroviruses have been identified to date, among which genogroup I (GI) and genogroup II (GII) noroviruses are major pathogens for humans. GI and GII noroviruses are further classified into nine and 27 genotypes, respectively. Noroviruses are well known to bind to histo‐blood group antigens (HBGAs). Many studies have revealed that virus‐like particles (VLPs) from different genotypes exhibit distinct patterns of HBGA binding, but the assay conditions used in these studies were not identical. To enable comparison of the binding to HBGA of nine GI genotypes, I purified VLPs from insect cells and analysed their HBGA‐binding profiles. Although each genotype exhibited a distinct pattern of HBGA binding, Lewis b antigen was commonly recognized by all of the genogroup I strains, suggesting that this antigen plays a critical role in the pathogenesis of noroviruses.

## Graphical abstract

Histo‐blood group antigens (HBGAs) are thought to be cellular attachment factors for human noroviruses. Using virus‐like particles from the genogroup I noroviruses, their HBGA‐binding profiles were investigated. Herein, I discovered that Lewis b antigen is a common ligand for all of the genogroup I strains, suggesting its critical role in the infection event.

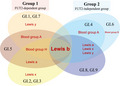

**Abbreviations**
BSA: bovine serum albumin
D‐PBS: Dulbecco's phosphate‐buffered saline
FUT2: the α1,2‐fucosyltransferase gene known as the secretor gene
Gal: galactose
GalNAc: *N*‐acetylgalactosamine
GI: genogroup I
GII: genogroup II
HBGA: histo‐blood group antigen
HSA: human serum albumin
LNDFH: lacto‐*N*‐difucohexaose
LNFP: lacto‐*N*‐fucopentaose
VLP: virus‐like particle

Noroviruses that belong to the family Caliciviridae are major causative agents of nonbacterial acute gastroenteritis in humans [1]. Ten genogroups of noroviruses have been identified to date, among which genogroup I (GI) and genogroup II (GII) noroviruses are major pathogens for humans. GI and GII noroviruses are further classified into 9 and 27 genotypes, respectively [2]. GII noroviruses account for almost 90% of norovirus incidents [3]. Among them, the genogroup II, genotype 4 (GII.4) viruses have been dominant for many years worldwide. A new GII.4 variant or subtype has been emerging every several years. The norovirus genome is a positive‐sense, single‐stranded RNA with a poly A tail in which three open reading frames (ORFs) are encoded. A genome‐linked viral protein, VPg, is thought to be bound at the 5′‐end of the genome. An ORF1 product is a polyprotein, which is eventually separated by an internal protease activity into six mature proteins. ORF2 and ORF3 encode major and minor structural proteins called VP1 (VP stands for a viral protein) and VP2, respectively.

A single norovirus virion is composed of 180 molecules of the VP1 capsid protein, and 90 dimers of VP1 proteins self‐assemble to form a *T* = 3 icosahedral particle [4]. Since the VP2 protein is abundant in basic amino acids, it has been suggested that the VP2 protein interacts with the RNA genome. When the norovirus ORF2 gene is expressed in insect cells via recombinant baculoviruses, virus‐like particles (VLPs) without the genome are formed and are usually excreted in culture media [5]. Normally, 38‐nm‐diameter VLPs are formed, but sometimes 23‐nm‐diameter VLPs are produced [6, 7]. The smaller particles have *T* = 1 icosahedral symmetry [8].

Noroviruses are well known to bind to histo‐blood group antigens (HBGAs) [9]. Especially, susceptibility to the GI.1 Norwalk strain is highly dependent on the secretor status; that is, the FUT2 null individuals are tolerant to infection by the Norwalk strain [10, 11, 12]. These earlier reports indicate that HBGAs are essential attachment factors, but their exact role in the infection process remains unclear. Many studies using VLPs and synthetic HBGAs have revealed that the VLPs from different genotypes exhibit distinct patterns of HBGA binding [13], but the assay conditions used in these studies were not identical, making it difficult to compare with the studies' results. Here I examined the HBGA binding of VLPs derived from nine genotypes classified in GI. The results clearly indicate that the Lewis b antigen is a common ligand for the GI noroviruses, suggesting that this antigen plays a critical role in the pathogenesis of noroviruses.

## Materials and methods

### Norovirus strains and plasmid construction

As summarized in Table 1, 10 strains belonging to different genotypes in GI were used in this study. The gene fragment carrying VP1 and VP2 proteins or VP1 alone was cloned into pORB baculovirus transfer vector (Allele Biotechnology, San Diego, CA, USA).

**Table 1:** Genogroup I norovirus strains used for the preparation of VLPs in this study.

Genotype	Strain	Accession number	Genes cloned into pORB transfer vector	Mutations introduced in VP1 protein^a^	References
GI.1	Seto	AB031013	VP1	V43A, A44P, T45V	[14]
GI.2	Funabashi 258	AB078335	VP1	V43A, A44P, T45V	[15, 16]
GI.3	Kashiwa 645	BD011871	VP1	A44P, T45V	[17]
GI.4	Chiba 407	AB042808	VP1 and VP2	M1K, M2K, L43A, A44P, T45V	[7, 8, 18]
GI.5	Siklos	KJ402295	VP1 and VP2	M1K, M2K, L43A, A44P, T45V	–
GI.6	WUG1	AB081723	VP1 and VP2	M1K, M2K, V43A, A44P, T45V	[19]
GI.7	Miyagi	AB758449	VP1 and VP2	None	[20]
	TCH‐060	JN005886	VP1 and VP2	M1K, M2K, A44P, T45V	[21]
GI.8	KY531	KJ196298	VP1 and VP2	M1K, M2T, V43A, A44P, T45V	–
GI.9	Vancouver 730	HQ637267	VP1 and VP2	None	[22]

^a^ The L43A/A44P/T45V triple mutation was originally described for the GI.4 Chiba strain in Ref. [7], which led to a homogeneous production of 38‐nm VLPs with an icosahedral symmetry of *T* = 3. A similar mutation was introduced in the VP1 proteins of some strains. The M1K/M2K or M1K/M2T mutation resulted in the elimination of the first two AUG codons from three consecutive AUGs and the translation initiation of VP1 from Met3, which effectively elevated the Chiba VLP yield when expressed in insect cells. The double mutation was also introduced in the GI.5 Siklos, GI.6 WUG1, GI.7 CH‐060 and GI.8 KY531 VP1 proteins.

### Preparation of VLPs

Sf9 insect cells (Oxford Expression Technologies, Oxford, UK) were transfected with the constructed pORB plasmids together with Sapphire Baculovirus DNA (Allele Biotechnology) to obtain recombinant baculoviruses, which were then used for VLP production in Trichoplusia ni cells (Oxford Expression Technologies). Typically, VLPs were collected from culture media and separated by isopycnic CsCl density gradient centrifugation. VLPs sedimented by ultracentrifugation were resuspended in distilled water unless otherwise stated and stored at 4 °C. For the preparation of the GI.4 Chiba VLPs, 20 mm sodium citrate buffer (pH 4.2) was used in place of distilled water. After preparation, the integrity of VLPs was evaluated by transmission electron microscopy with uranyl acetate used as a stain (Fig. S1).

### Antisera

The rabbit antisera reacted with the GI.1, GI.2, GI.3, GI.4 or GI.6 strains were laboratory stocks. The rabbit anti‐GI.9 VLP antiserum was prepared by Scrum, Inc. (Tokyo, Japan) [22]. For the detection of the GI.5, GI.7 and GI.8 VLPs, a mixture of the above six antisera was used.

### HBGA binding assay

The HBGA binding assay was performed as described previously [22]. The bovine serum albumin (BSA) or human serum albumin (HSA) conjugates of various HBGAs were purchased from Dextra Laboratories Ltd (Reading, UK) or IsoSep (Tullinge, Sweden) and listed in Table 2. The symbolic structures of the HBGAs used are depicted according to the standardized way of drawing [23] for clarity in Fig. S2. BSA (FUJIFILM Wako Pure Chemical Corporation, Osaka, Japan), HSA (Nacalai Tesque, Kyoto, Japan), the lactose‐BSA conjugates (Sigma, St. Louis, MO, USA) and the lactose‐HSA conjugate (IsoSep) were used as negative controls.

**Table 2:** HGBA conjugates used in this study.

HBGA conjugates	Manufacturer	Cat. number	Numbers of saccharides	Average sugar residues per protein molecule	Spacer between vsugar and BSA/HSA	Type of the linkage of the terminal Gal‐GalNAc^a^
Blood group A‐BSA	Dextra	NGP6305	3	17	6 atoms	na^b^
Blood group B‐BSA	Dextra	NGP0323	3	13	3 atoms	na^b^
Lewis x‐BSA	Dextra	NGP0302	3	18	3 atoms	Type 2
LNFP I‐BSA	Dextra	NGP0503	5	20	3 atoms	Type 1
LNFP II‐BSA	Dextra	NGP0501	5	15	3 atoms	Type 1
LNFP III‐BSA	Dextra	NGP0502	5	15	3 atoms	Type 2
LNDFH I‐BSA	Dextra	NGP0601	6	7.5	3 atoms	Type 1
Lewis y‐HSA	IsoSep	60/95	4	13.3	APE (aminophenylethyl)	Type 2

^a^ Some HBGAs have the terminal galactosyl‐*N*‐acetylgalactosamine disaccharide, in which the galactose moiety (Gal) is connected to the *N*‐acetyl galactosamine (GalNAc) with type 1 (β1–3) or type 2 (β1–4) linkage.

^b^ na, not applicable. These conjugates do not contain the GalNAc moiety.

**Fig. 1:**
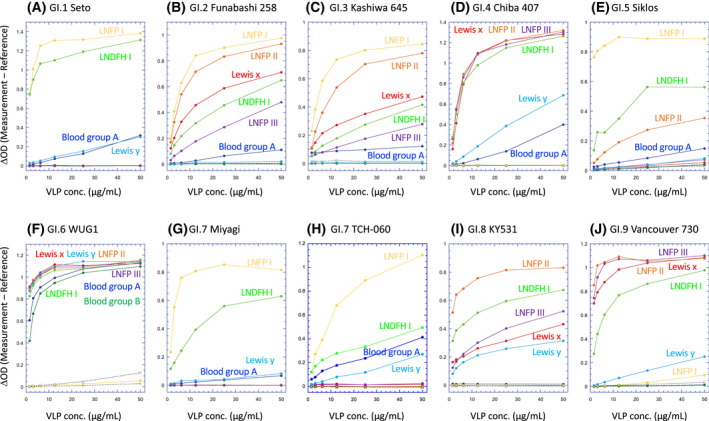
HBGA binding assay of VLPs. The HBGA binding assay was done as described previously [22] for the GI.1 Seto (A), GI.2 Funabashi 258 (B), GI.3 Kashiwa 645 (C), GI.4 Chiba 407 (D), GI.5 Siklos (E), GI.6 WUG1 (F), GI.7 Miyagi (G), GI.7 TCH‐060 (H), GI.8 KY531 (I) and GI.9 Vancouver 730 (J) VLPs prepared from T. ni insect cells. The commercially available conjugates of the ABH and Lewis antigens with BSA or HSA were used, and are represented by colors with the name of the HBGA on the respective panels. The lactose‐BSA (open circle with a dotted line) and lactose‐HSA (open square with a dotted line) conjugates were used as negative controls. Each of the lacto‐series (having the type 1 linkage) and neolacto‐series (having the type 2 linkage) BSA conjugates includes the following oligosaccharides: LNFP I, Pentasaccharide containing blood group H type 1 trisaccharide (yellow); LNFP II, Pentasaccharide containing Lewis a trisaccharide (orange); LNFP III, Pentasaccharide containing Lewis x trisaccharide (purple); LNDFH I, Hexasaccharide contanning Lewis b tetrasaccharide (yellow green). Blood groups A (blue) and B (green), and Lewis x (red) conjugates include the respective terminal trisaccharides, and the Lewis y conjugate includes the terminal tetrasaccharide. A graph for the GI.9 Vancouver VLP was redisplayed from Ref. [22] for comparison. [Colour figure can be viewed at wileyonlinelibrary.com]

For the determination of the VLPs' preferences for HBGAs in an objective way, the data in Table S1 were summarized from the following viewpoint. With the use of the kaleida graph software (Hulinks, Tokyo, Japan), I evaluated whether the data of the HBGA binding assay for VLPs (Fig. 1) could be fit to the Michaelis–Menten equation [24] below.

(1) $$v=\frac{V_{\max}\left[S\right]}{K_{m}+\left[S\right]}.$$

Considering the experiments done in this study together with the Hill equation [25], the terms “ligand” and “receptor” correspond to VLPs added and HBGAs fixed on the microplate, respectively. Therefore, the equation, Eqn (1), should thus be regarded as follows:

(2) $$\Delta \mathrm{OD}=\frac{\Delta {\mathrm{OD}}_{\max}\left[\mathrm{VLP}\right]}{{\mathrm{VLP}}_{50}+\left[\mathrm{VLP}\right]}.$$

Here, the variables ΔOD and [VLP] represent the vertical and transverse values, respectively, for the graphs shown in Fig. 1. VLP_50_ can be considered the concentration of VLPs when 50% of the HBGAs are captured by VLPs, and ΔOD will reach ΔOD_max_ when all HBGAs are occupied by VLPs.

When [*S*] is much lower than the *K*_m_ value ([*S*] ≪ *K*_m_), the Michaelis–Menten equation, Eqn (1), can be modified to the following approximation.

(3) $$v=\frac{V_{\max}}{K_{m}}\left[S\right].$$

This equation means that the reaction rate is proportionate to the substrate concentration, so that the larger the constant *V*_max_/*K*_m_ is, the faster the turnover rate of the enzyme is. Similarly, considering the binding between VLPs and HBGAs, the ΔOD_max_/VLP_50_ value will represent the efficiency of the binding.

The constants ΔOD_max_ and VLP_50_ in Table S1, were calculated by the kaleida graph software with standard errors, and then the ΔOD_max_/VLP_50_ values were determined. For some combinations of VLP and HBGA, the plots of [VLP] versus ΔOD did not exhibit a significant hyperbolic shape, and the VLP_50_ value was much larger than the highest concentration of VLPs used in the experiment. The values were therefore considered less reliable, as indicated by parentheses and “a” in Table S1. In addition, the ΔOD_max_ values were also abnormally high in a few cases, which is considered unreliable.

Note that the ΔOD values depended on the reaction between VLPs and antibodies, and therefore the ΔOD values obtained for VLPs from one strain cannot simply be compared with those obtained for VLPs from the other strain. The ΔOD values should be evaluated for the preference of HBGAs against VLPs from a single strain. These parameters for the VLP‐HBGA interaction are thought to reflect the avidity of VLPs for HBGAs.

## Results and Discussion

### Preparation of VLPs

Virus‐like particles from the nine strains (GI.1 Seto, GI.2 Funabashi, GI.3 Kashiwa, GI.4 Chiba, GI.6 WUG1, GI.7 Miyagi, GI.7 TCH‐060, GI.8 KY531 and GI.9 Vancouver) could be collected from culture media of the T. ni insect cells, and these VLPs were morphologically homogeneous, showing typical 38‐nm diameter particles, as indicated in our previous reports [7, 8, 21, 22]. In contrast, VLPs from the GI.5 Siklos strain were collected from water‐extract of the recombinant baculovirus‐infected T. ni cells, since these particles were rarely excreted into media.

### HBGA binding profile of the nine GI genotypes

For the determination of the HBGA‐binding profiles of VLPs from the nine different GI genotypes mentioned above, the binding assay was performed using commercially available BSA or HSA conjugates of HBGAs. As shown in Fig. 1, it is clear that (a) the binding profiles of the VLPs are not the same for all genotypes and (b) the extent of binding varies with the combinations of VLPs and HBGAs. It is to be noted that it is difficult to compare the degree of HBGA binding between different VLPs since a common, equally reactive, primary antibody is not available for all strains tested in the HBGA binding assay. However, it would be enough to know which HBGA is preferentially bound to VLPs from a specific norovirus strain.

The binding patterns of each genotype are summarized in Table 3 and Table S1. Based on these results, the HBGA‐binding pattern was categorized into the two major groups. Group 1 includes GI.1, GI.2, GI.3, GI.5 and GI.7, whose HBGA binding is dependent on the FUT2 secretor gene encoding a fucosyltransferase, and group 2 includes GI.4, GI.6, GI.8 and GI.9, whose HBGA binding is not dependent on the secretor gene. Group 1 is further divided into the following three subgroups. Subgroup 1a; GI.1 and GI.7 strains prefer the H antigen the most, with the Lewis b antigen in the second place. These strains also significantly bind to the A antigen and Lewis y antigens but do not bind to the B antigen or the Lewis a and x antigens. Subgroup 1b; GI.2 and GI.3 strains bind to various types of HBGAs with the exceptions of the B and Lewis y antigens. The order of preference for other HBGAs is identical for these two genotypes. Subgroup 1c; GI.5 strain binds to the Lewis a and b antigens which have the type 1 linkage, but not the Lewis x and y antigens with the type 2 linkage. Therefore, it is plausible that this strain binds to LNFP I that includes the H epitope connected via the type 1 linkage. Group 2 is further divided into the following two subgroups. Subgroup 2a; GI.4 and GI.6 strains bind to all Lewis antigens and the A antigen, although the degree of binding to some HBGAs differs between these two strains. It is surprising that GI.6 is the only genotype in GI that binds to the B antigen. The GI.4 and GI.6 strains do not bind to the H antigen. Subgroup 2b; The HBGA profiles of GI.8 and GI.9 strains are similar to each other. These two strains prefer binding to all of the Lewis antigens. It might not be significant that the GI.9 strain weakly binds to the H antigen.

**Table 3:** The HBGA binding profiles of each of the genogroup I noroviruses. This table is summarized on the basis of the data shown in Table S1. The intensity of the VLP‐HBGA binding is shown with marks such as “+++”, “++”, “+”, or “−”, which represents the maximum to minimum binding intensity in this order: +++, the ΔOD_max_/VLP_50_ value is ≥ 0.1; ++, ≥ 0.01 and < 0.1; +, < 0.01; −, no binding is observed.

Genotype	Strain	Blood group H (LNFP I, Lewis d)	Blood group A	Blood group B	Lewis a (LNFP II)	Lewis x (LNFP III)	Lewis b (LNDFH I)	Lewis y
GI.1	Seto	+++	+	−	−	−	+++	+
	Norwalk^a^	[+]	[+]	[−]	[−]	[−]	[+]	[+]
GI.2	Funabashi 258	+++	+	−	+++	++	++	−
GI.3	Kashiwa 645	+++	+	−	+++	++	++	−
GI.4	Chiba 407	−	+	−	+++	+++	+++	++
GI.5	Siklos	+++	+	−	++	−	++	−
GI.6	WUG1	−	+++	+++	+++	+++	+++	+++
GI.7	Miyagi	+++	+	−	−	−	++	+
	TCH‐060	+++	++	−	−	−	++	+
	TCH‐060^a^	[+]	[+]	[−]	[+]	[+]	n/a	[+]
GI.8	KY531	−	−	−	+++	++	+++	++
	Boxer^a^	[−]	[−]	[−]	[+]	[−]	[+]	[+]
GI.9	Vancouver 730	+	−	−	+++	+++	+++	+

^a^ As for the GI.1 Norwalk [27, 28], GI.7 TCH‐060 [21] and GI.8 Boxer [29] strains, it is indicated whether the binding was achieved ([+]) or not ([−]). “n/a”, the data were not available.

Most importantly, VLPs from all of the genotypes bind to the Lewis b antigen, suggesting that this HBGA plays a critical role in norovirus infection; this reflects the similar finding that the microaerophilic bacterium *Helicobacter pylori* recognizes the Lewis b antigen for infection [26]. It is also notable that the two strains in each of Subgroups 1a, 1b, and 2a are not always genetically related to each other according to a recent phylogenetic analysis [2].

Regarding the GI.1 Norwalk [27, 28], GI.2 Funabashi 258 [16], GI.7 TCH‐060 [21], GI.8 Boxer [29] and GI.9 Vancouver [22] strains, the crystal structures of the HBGA complexes of the respective VP1 proteins were resolved, and parts of their HBGA binding profiles were known. The profile of the Seto strain is consistent with that of the Norwalk strain [13, 27, 28], which is supported by the fact that these two VP1 proteins are highly homologous (98% amino acid identity and 100% homology). On the other hand, there are discrepancies between the previous reports and the present findings for GI.7 and GI.8. As for GI.8, the Miyagi strain binds to the Lewis x antigen, but binding for the Boxer strain was not observed in the previous investigation [29]. This might be because of the HBGA conjugate used for the binding assay. For example, I obtained seemingly contradictory results that any VLPs prepared in this study did not bind to Lewis a trisaccharide‐BSA conjugates (data not shown) although they bound to the BSA conjugates of LNFP II including the Lewis a trisaccharide moiety in its terminal. This observation suggests that, in some commercially available conjugates, oligosaccharide moieties are not presented in an adequate manner for VLPs to bind. It is possible that the Lewis x conjugate (Lewis x trisaccharide‐biotin conjugate) used in the study of the GI.8 Boxer strain [29] was not adequate for the binding assay.

In contrast, there was a marked difference among the GI.7 strains. Shanker et al. [21] reported that the TCH‐060 VLP could bind to the Lewis a antigen and determined crystal structures of its P domain with Lewis a, x or y antigens. However, in my *in vitro* biding assay, neither the Miyagi VLP nor the TCH‐060 VLP could bind to Lewis a and x antigens. No binding was observed although the VLPs reacted with HBGAs at 4 °C (data not shown), as was observed for the TCH‐060 VLPs [21]. VP1 proteins from these two strains are highly homologous, as indicated by their 99% amino acid identity (100% homology). This would be well reflected by the result that observed binding profiles for these two strains are the same although there were slight differences as to the level of binding to blood group A antigen and Lewis b and y antigens (Fig. 1). Such differences might be caused by subtle differences in conditions of VLPs themselves which could be varied by preparation; for example, slight aggregation of VLPs might affect the accessibility to HBGAs even if their morphology was observed similarly by electron microscopy. The discrepancy between this result and the previous result [21] of HBGA binding assay would rather be attributable to the difference in HBGA conjugates (BSA‐conjugate vs. biotin‐conjugate). In the present study, no biotin‐conjugates of HBGAs were tested. Another possibility is that amino acid residues that differ between the two GI.7 VP1 proteins are critical for the recognition of the Lewis a and x antigens. However, an amino acid alignment indicated that all amino acids involved in HBGA binding in the TCH‐060 VP1 protein [21] are conserved in the Miyagi counterpart. Thus, this would be not the case. As described in the previous report from my group [22], there is a limitation because the *in vitro* HBGA binding assay may not have precisely revealed all of the characteristics of VLPs. There is no doubting that the TCH‐060 VP1 protein were complexed with Lewis a and x antigens [21]. Considering the result in this study, it is assumed that avidity of these GI.7 VP1 proteins for Lewis a and x antigens is not so high. Therefore, I might not be able to detect any binding of GI.7 VLPs to these antigens.

It is also notable that the GI.6 VLPs bind to a wide variety of HBGAs (Fig. 1F) and that GI.6 is the only genotype capable of binding to blood group B antigen. Structural analyses will be required to understand the molecular mechanism for that unexpected recognition. Currently, although large outbreaks evoked by the GI.6 noroviruses have not been reported worldwide, their broad HBGA recognition similar to that observed for GII.4 strains warrants epidemiological monitoring for potential emergence as pandemic strains.

### Physiological and biological roles of Lewis antigens in norovirus infection

Although there is some variation in the binding profiles, binding to Lewis antigens is likely a common characteristic of the GI noroviruses [13]. The results described above clearly support this idea and further indicate that the Lewis b antigen is a common ligand for the GI noroviruses. Several reports have noted that Lewis antigens are expressed in normal intestinal tissues, and that their expression patterns vary among individuals, and the expression of Lewis antigens is sometimes inconsistent with the individuals' secretor statuses [30, 31, 32]. In any case, it is possible that Lewis b‐positive individuals are infected by the GI noroviruses.

The following question thus arises: what is the role of HBGAs including the Lewis b antigen in the process of norovirus infection? For many years, HBGAs have been assumed to be receptors for noroviruses. Since HBGAs actually affect humans' susceptibility to some genotypes of noroviruses [9, 10, 11, 12], we cannot rule out the idea that HBGAs are principal receptors for infection. It would be worthwhile to determine whether HBGA binding induces morphological changes of a norovirus virion, as has been observed for the receptor‐bound feline calicivirus particle [33].

## Conflict of interest

The author declares no conflict of interest.

## Author contributions

YS designed and performed experiments, analyzed data and wrote the manuscript.

## Supporting information


**Fig. S1.** Transmission electron microscopic images of VLPs prepared from insect cell culture. VLPs were prepared as described under the Materials and Methods in the main text and stained with uranyl acetate for taking images. The bars indicate 200 nm, except for panel F, in which it indicates 100 nm.

**Fig. S2.** Symbolic representation of the HBGAs used for the binding assay.

**Table S1.** The parameters for the binding between VLPs and HBGAs.

## Data Availability

The data that support the findings of this study are available on request from the corresponding author. The data are not publicly available due to privacy restrictions.
