# Supplementary material for: Lewis b antigen is a common ligand for genogroup I norovirus strains
Source: FEBS Open Bio. 2022 Jul 4;12(9):1688–95. doi: 10.1002/2211-5463.13455 (PMC9433824; doi:10.1002/2211-5463.13455)
Supplement: Supplementary file 1 — Fig. S1. Transmission electron microscopic images of VLPs prepared from insect cell culture. VLPs were prepared as described under the Materials and Methods in the main text and stained with uranyl acetate for taking images. The bars indicate 200 nm, except for panel F, in which it indicates 100 nm. [file FEB4-12-1688-s003.pdf]

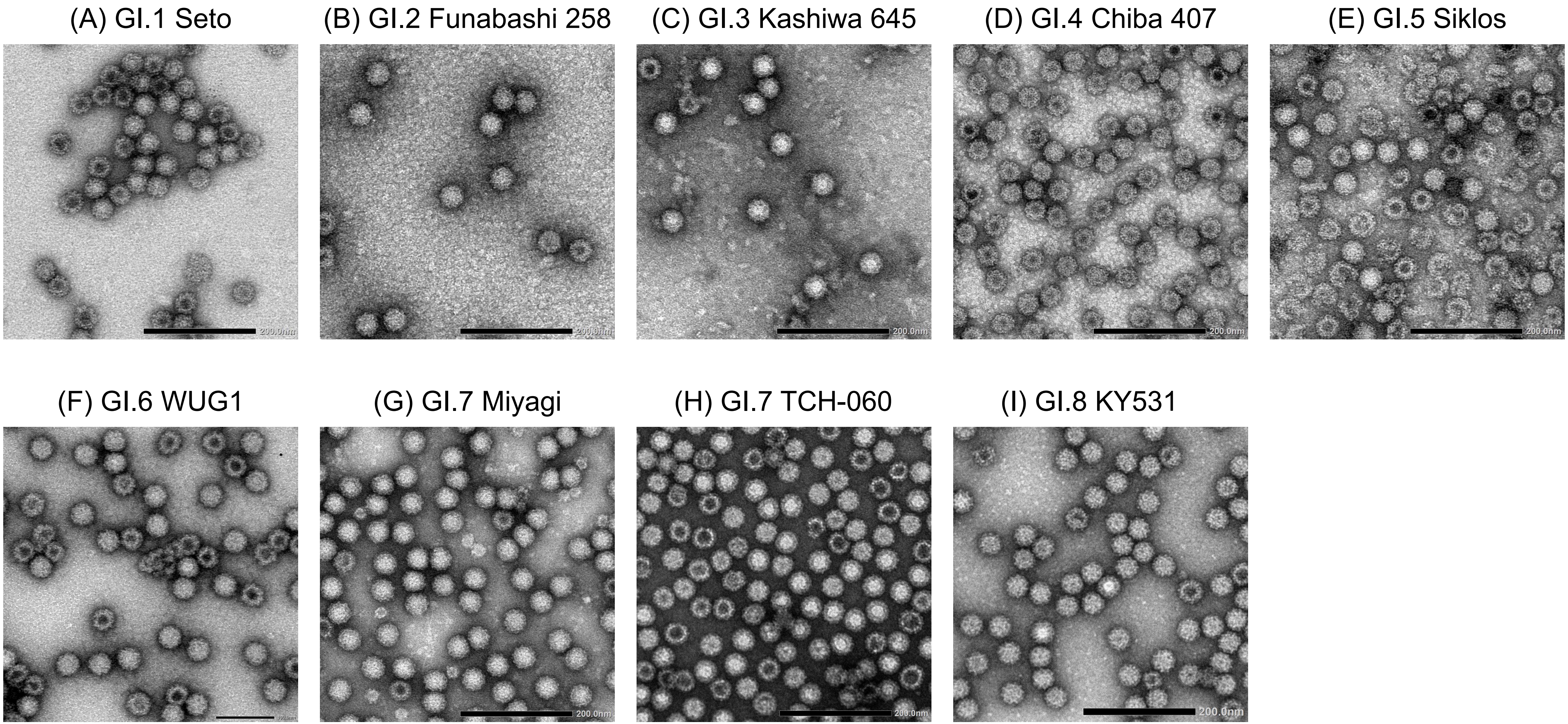


**Supplementary figure 1. Transmission electron microscopic images of VLPs prepared from insect cell culture.** VLPs were prepared as described under the Materials and Methods in the main text, and stained with uranyl acetate for taking images. The bars indicate 200 nm, except for panel F, in which it indicates 100 nm.
