# Supplementary material for: Lewis b antigen is a common ligand for genogroup I norovirus strains
Source: FEBS Open Bio. 2022 Jul 4;12(9):1688–95. doi: 10.1002/2211-5463.13455 (PMC9433824; doi:10.1002/2211-5463.13455)
Supplement: Supplementary file 2 — Fig. S2. Symbolic representation of the HBGAs used for the binding assay. [file FEB4-12-1688-s002.pdf]

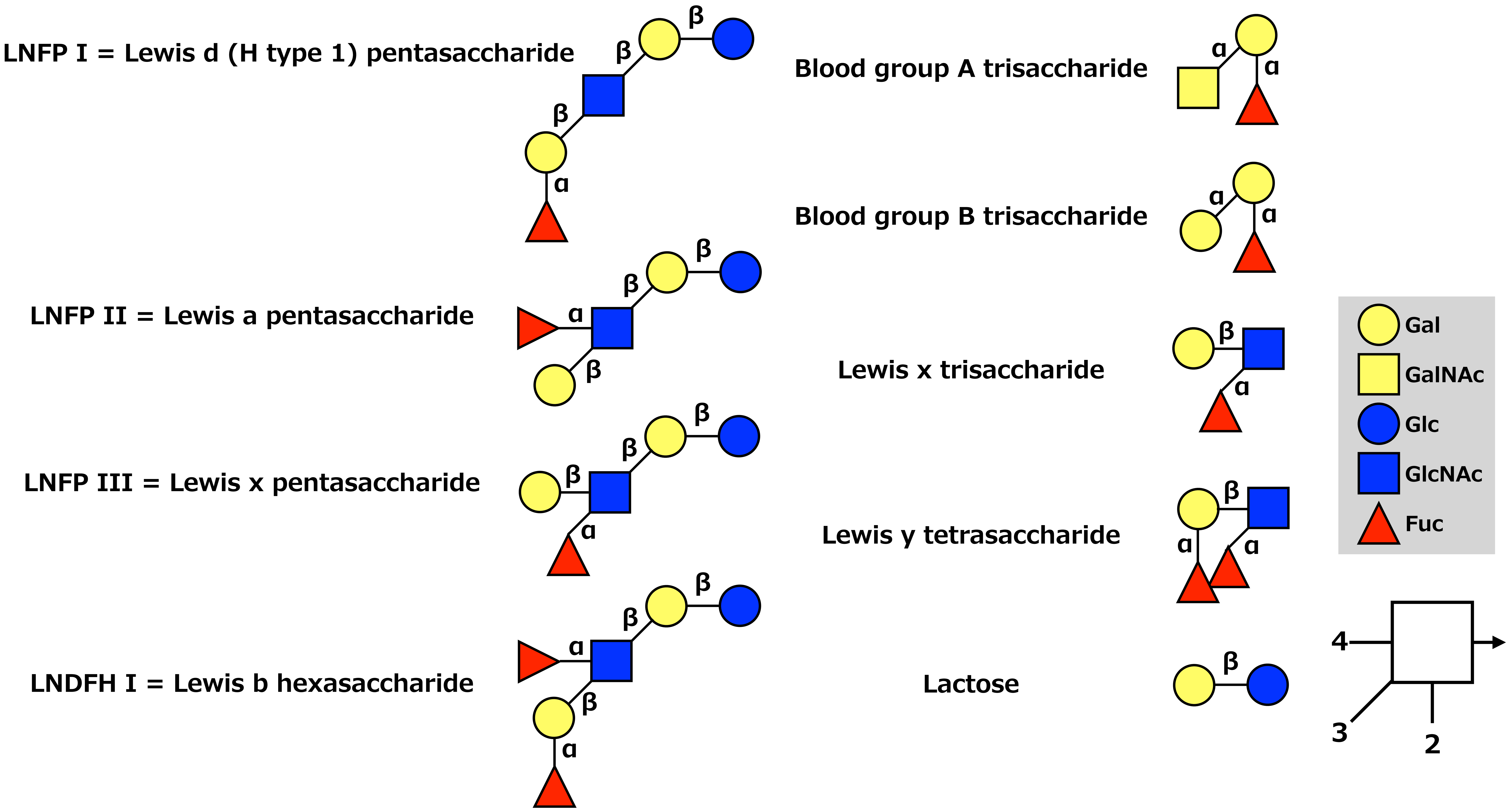

Supplementary Figure 2. Symbolic representation of the HBGAs used for the binding assay.
