# Supplementary material for: Lewis b antigen is a common ligand for genogroup I norovirus strains
Source: FEBS Open Bio. 2022 Jul 4;12(9):1688–95. doi: 10.1002/2211-5463.13455 (PMC9433824; doi:10.1002/2211-5463.13455)
Supplement: Supplementary file 3 — Table S1. The parameters for the binding between VLPs and HBGAs. [file FEB4-12-1688-s001.docx]

**Supplementary Table 1. The parameters for the binding between VLPs and HBGAs.**

| Genotype and strain | | Parameters | LNFP I-BSA | Blood group A-BSA | Blood group B-BSA | LNFP II-BSA | LNFP III-BSA | Lewis x-BSA | LNDFH I-BSA | Lewis y-HSA |
| --- | --- | --- | --- | --- | --- | --- | --- | --- | --- | --- |
|  |  |  | H type 1 penta | A tri | B tri | Lewis a penta | Lewis x penta | Lewis x tri | Lewis b hexa | Lewis y tetra |
| GI.1 | Seto | ΔOD_max_ | 1.43 ± 0.03 | 0.80 ± 0.44 | – | – | – | – | 1.27 ± 0.04 | 1.35 ± 0.74 |
|  |  | VLP_50_ (µg/mL) | 1.32 ± 0.17 | (131 ± 83) ^a^ | – | – | – | – | 1.20 ± 0.20 | (176 ± 119) ^a^ |
|  |  | ΔOD_max_ / VLP_50_ | 1.1 | (0.0061) | – | – | – | – | 1.1 | (0.0077) |
| GI.2 | Funabashi 258 | ΔOD_max_ | 1.11 ± 0.05 | 1.06 ± 1.04 | – | 1.06 ± 0.03 | 1.11 ± 0.14 | 0.84 ± 0.02 | 0.90 ± 0.08 | – |
|  |  | VLP_50_ (µg/mL) | 5.15 ± 0.84 | (424 ± 456) ^a^ | – | 6.63 ± 0.61 | 67.0 ± 12.5 | 10.1 ± 0.58 | 21.2 ± 4.14 | – |
|  |  | ΔOD_max_ / VLP_50_ | 0.22 | (0.0025) | – | 0.16 | 0.017 | 0.084 | 0.042 | – |
| GI.3 | Kashiwa 645 | ΔOD_max_ | 0.94 ± 0.03 | 0.11 ± 0.01 | – | 0.96 ± 0.03 | 0.43 ± 0.10 | 0.53 ± 0.05 | 0.64 ± 0.09 | – |
|  |  | VLP_50_ (µg/mL) | 4.19 ± 0.45 | 0.90 ± 0.52 | – | 10.0 ± 0.77 | 30.8 ± 13.7 | 9.47 ± 2.48 | 29. 3 ± 8.05 | – |
|  |  | ΔOD_max_ / VLP_50_ | 0.22 | 0.12 | – | 0.095 | 0.014 | 0.056 | 0.022 | – |
| GI.4 | Chiba 407 | ΔOD_max_ | – | (3.0 x 10^12^ ± 4.7 x 10^15^) ^a^ | – | 1.49 ± 0.09 | 1.46 ± 0.07 | 1.54 ± 0.13 | 1.40 ± 0.04 | (4.72 ± 1.55) ^a^ |
|  |  | VLP_50_ (µg/mL) | – | (4.1 x 10^14^ ± 3.4 x 10^17^) ^a^ | – | 5.25 ± 1.03 | 5.03 ± 0.79 | 6.87 ± 1.75 | 5.35 ± 0.49 | (292 ± 109) ^a^ |
|  |  | ΔOD_max_ / VLP_50_ | – | (0.0073) | – | 0.28 | 0.29 | 0.22 | 0.26 | (0.016) |
| GI.5 | Siklos | ΔOD_max_ | 0.90 ± 0.01 | 0.27 ± 0.09 | – | 0.47 ± 0.02 | – | – | 0.65 ± 0.08 | – |
|  |  | VLP_50_ (µg/mL) | 0.30 ± 0.04 | 44.7 ± 25.4 | – | 17.6 ± 1.76 | – | – | 7.26 ± 2.62 | – |
|  |  | ΔOD_max_ / VLP_50_ | 3.02 | 0.0061 | – | 0.027 | – | – | 0.09 | – |
| GI.6 | WUG1 | ΔOD_max_ | – | 1.13 ± 0.02 | 1.15 ± 0.02 | 1.12 ± 0.02 | 1.12 ± 0.02 | 1.13 ± 0.01 | 1.12 ± 0.02 | 1.15 ± 0.01 |
|  |  | VLP_50_ (µg/mL) | – | 1.37 ± 0.12 | 2.42 ± 0.18 | 0.44 ± 0.07 | 0.43 ± 0.09 | 0.41 ± 0.05 | 0.61 ± 0.08 | 0.55 ± 0.06 |
|  |  | ΔOD_max_ / VLP_50_ | – | 0.83 | 0.47 | 2.6 | 2.6 | 2.7 | 1.9 | 2.1 |
| GI.7 | Miyagi | ΔOD_max_ | 0.93 ± 0.07 | 0.18 ± 0.06 | – | – | – | – | 0.80 ± 0.04 | 0.13 ± 0.05 |
|  |  | VLP_50_ (µg/mL) | 2.52 ± 0.86 | (82.4 ± 40.2) ^a^ | – | – | – | – | 12.5 ± 1.74 | 35.6 ± 12.3 |
|  |  | ΔOD_max_ / VLP_50_ | 0.37 | (0.0021) | – | – | – | – | 0.064 | 0.0037 |
| GI.7 | TCH-060 | ΔOD_max_ | 1.44 ± 0.06 | 0.64 ± 0.15 | – | – | – | – | 0.52 ± 0.07 | (17.0 ± 170) ^a^ |
|  |  | VLP_50_ (µg/mL) | 15.0 ± 1.5 | 31.7 ± 14.1 | – | – | – | – | 8.31 ± 3.19 | (3.1 x10^3^ ±  3.2 x 10^4^) ^a^ |
|  |  | ΔOD_max_ / VLP_50_ | 0.10 | 0.020 | – | – | – | – | 0.063 | (0.0055) |
| GI.8 | KY531 | ΔOD_max_ | – | – | – | 0.83 ± 0.02 | 0.59 ± 0.05 | 0.41 ± 0.05 | 0.65 ± 0.04 | 0.34 ± 0.02 |
|  |  | VLP_50_ (µg/mL) | – | – | – | 1.01 ± 0.12 | 9.99 ± 2.56 | 4.59 ± 2.12 | 2.21 ± 0.58 | 6.34 ± 1.03 |
|  |  | ΔOD_max_ / VLP_50_ | – | – | – | 0.82 | 0.06 | 0.089 | 0.29 | 0.053 |
| GI.9 | Vancouver 730 | ΔOD_max_ | (5.9 x 10^16^ ± 4.1 x 10^23^) ^a^ | – | – | 1.10 ± 0.02 | 1.12 ± 0.02 | 1.07 ± 0.03 | 1.04 ± 0.02 | 1.94 ± 0.17 |
|  |  | VLP_50_ (µg/mL) | (3.4 x 10^19^ ± 2.4 x 10^26^) ^a^ | – | – | 0.39 ± 0.08 | 0.74 ± 0.08 | 0.97 ± 0.15 | 4.45 ± 0.25 | 337 ± 33 |
|  |  | ΔOD_max_ / VLP_50_ | (0.0017) | – | – | 2.8 | 1.5 | 1.1 | 0.23 | 0.0058 |

The parameters were calculated by fitting the plots of [VLP] vs. ΔOD to the Michaelis Menten equation, where [VLP] was regarded as the substrate concentration ([S]), and ΔOD was regarded as the reaction rate (v). tri: trisaccharide, tetra: tetrasaccharide, penta: pentasaccharide, hexa: hexasaccharide.

^a^ The plots of [VLP] vs. ΔOD were not hyperbolic, and thus reliable values could not be obtained.
